# Translation, transcultural adaptation, and validation of the role-modeling cost-conscious behaviors scale

**DOI:** 10.1186/s12909-019-1587-x

**Published:** 2019-05-16

**Authors:** Marta Silva Menezes, Marília Menezes Gusmão, Rui Nei de Araújo Santana, Carolina Villa Nova Aguiar, Dilton Rodrigues Mendonça, Rinaldo Antunes Barros, Mary Gomes Silva, Liliane Lins-Kusterer

**Affiliations:** 1 School of Medicine, Bahiana School of Medicine and Public Health, Salvador, Bahia Brazil; 2 School of Psychology, Bahiana School of Medicine and Public Health, Salvador, Bahia Brazil; 3 School of Nursing, Bahiana School of Medicine and Public Health, Salvador, Bahia Brazil; 4 School of Medicine, Federal University of Bahia, Praça XV de Novembro, Largo do Terreiro de Jesus s/n, Salvador, Bahia CEP 400260-10 Brazil

**Keywords:** Medical professionalism, Costs, health care, Education, medical, Rating scale, behavioral, Students, medical

## Abstract

**Background:**

Training in the use of cost-conscious strategies for medical students may prepare new physicians to deliver health care in a more sustainable way. Recently, a role-modeling cost-conscious behaviors scale (RMCCBS) was developed for assessing students’ perceptions of their teachers’ attitudes to cost consciousness. We aimed to translate the RMCCBS into Brazilian Portuguese, adapt the scale, transculturally, and validate it.

**Methods:**

We adopted rigorous methodological approaches for translating, transculturally adapting and validating the original scale English version into Brazilian Portuguese. We invited all 400 undergraduate medical students enrolled in the 5th and 6th years of a medical course in Northeast Brazil between January and March 2017 to participate. Of the 400 students, 281 accepted to take part in the study. We analyzed the collected data using the SPSS software version 21 and structural equation modeling (SEM) was performed using AMOS SPSS version 18. We conducted exploratory factor analysis (EFA), varimax rotation, with Kaiser Normalization and Principal Axis Factoring extraction method. We conducted confirmatory factor analysis (CFA), using the SEM. We used the following indexes of adherence of the model: Comparative fit index (CFI), Goodness-of-fit index (GFI) and Tucker-Lewis Index (TLI). We considered the Bayesian Information Criterion (BIC) for Sample-size adjusted. The root mean square error of approximation was calculated. Values below 0.08 were considered acceptable. Composite reliability analyzes were performed to evaluate the accuracy of the instrument. Values above 0.70 were considered satisfactory.

**Results:**

Of the 281 undergraduate medical students, 195 (69.3%) were female. Mean age of participants was 25.0 ± 2.6 years. In the EFA, the KMO was 0.720 and the Bartlett sphericity test was significant (*p* < 0.001). We conducted the EFA into two factors: role-modeling cost-conscious behaviors in health (seven items) and health waste behaviors (six items). The 13 item-scale was submitted to composite reliability analyzes, obtaining values of 0.813 and 0.761 for the role-modeling cost-conscious behaviors and the health waste behaviors factors, respectively.

**Conclusions:**

We concluded that the cost-conscious behaviors scale has good psychometric properties and is a valid and reliable instrument for evaluating medical students’ perception of their teachers’ cost-conscious behaviors.

## Background

The exponential increase in health costs should be a priority concern for governments, health professionals, healthcare companies, physicians, and patients [1]. Not only demographic and socioeconomic factors such as age, health status, and income increase public healthcare expenditure each year [1], but also preventable hospital admissions, inappropriate treatment and misuse of diagnostic testing.

Exercising cost-consciousness always represents a challenge for Physicians. Besides dealing with private gain, personal advantages and other potential conflicts of interests, their responsibilities include the wisely use of health care resources, as well as the duty to guarantee the patients’ best interests [2, 3]. In a situation of pressure from patients to order more tests, it is also difficult for the physician to maintain the right cost-conscious behavior [4–6]. To maintain the base of medicine’s social contract, in times of conflict of interests, physicians need continuous education, exercising the principles of professionalism, enhancing their personal commitment to patients’ welfare and collectivity. Undergraduate and post-undergraduate medical education may help future physicians to address health care costs [7–10].

A randomized study in the USA included 2556 physicians, representing many medical specialties and all currently practicing, showed that only 36% believed that practicing physicians had major responsibility for reducing health care costs. Most of them reported lawyers (60%), health insurance companies (59%), health systems (56%), pharmaceutical industries (56%) and patients (52%) as having the main responsibility to reduce costs in health care [11]. Another representative study with resident doctors suggests that the training environment may have a later influence on the physicians’ cost-consciousness [12]. Authors suggest the improvement of medical education training in order to promote cost-conscious behaviors.

Approaches using cost-conscious strategies during the training of medical students and residents may be effective, and medical students seem to be more accepting of cost-conscious care than physicians [10]. In Brazil, it is also important to implement institutional measures to allow the continued development of medical teachers in terms of knowledge, skills, and innovation in medical education, since the Brazilian system of medical education adopted physicians as medical student preceptors, mainly in public health services for both private and public medical schools [13]. The improvement and development of formal and informal curricula is a fundamental basis of teaching cost-consciousness preparing new physicians to deliver health care in a sustainable way for the future [14].

Recent studies have pointed out that training measures of high-value cost-conscious care, during the medical learning phases, influence physicians’ practice behaviors during their career. Medical school and residency programs should continue to collaborate in the development of curricula promoting the early exposure of medical students to the practice of cost-conscious behavior [8, 14–18]. A recent systematic review suggests that the combination of specific knowledge transmission, reflective practice, and a supportive environment produces a better medical education and high values in cost-conscious care [19].

Several theoretical frameworks and empirical research have been implemented to evaluate professionalism and cost-conscious attitudes of physicians [11, 19–23]. In a recent study based on previous questionnaires, a role-modeling cost-conscious behaviors scale was developed for assessing students’ perceptions of their teachers’ attitudes of cost consciousness [8]. This scale may be used as a medical education tool for enhancing cost-conscious practice during medical training. We have no similar validated instrument in Brazil, therefore we translated the role-modeling cost-conscious behaviors scale (RMCCBS) into Brazilian Portuguese, adapted it transculturally and validated it.

## Methods

### Instrument

The role-modeling cost-conscious behaviors scale was proposed by Leep Hunderfund et al. [8]. This scale is composed of 13 items distributed in two domains: cost-conscious health behaviors (seven items) and health waste behaviors (six items). We asked medical students to rate themselves on each item, using a Likert 4-point scale from 1 (totally disagree) to 4 (totally agree).

### Translation

The RMCCBS scale was translated from the original English into Brazilian Portuguese by two independent translators, fluent in English. The two translators were from distinct backgrounds. The first translator had knowledge of the health area and of the content area of the scale. The second translator did not have knowledge of medical terminology. A third translator participated in the syntheses of the two translated versions, containing both medical and usual spoken language, and they elaborated a single consensual version.

A back-translation into English was carried out by a native English speaker and teacher. The back-translated version and the original English version were compared by another native English speaker for evaluation of whether the text preserved its original meaning and by a panel of specialists.

We applied the preliminary version of the scale to 11 medical students in their 3rd year of the undergraduate course. They answered the questionnaire and evaluated item comprehension for semantic validation. The suggestions made by these students were submitted to another panel of researchers comprised of four professors and three medical students [24, 25].

### Sampling

We invited all 400 undergraduate medical students, in their fifth and sixth year of the medical course at a private medical school, to participate in the study. Data were collected between January and March 2017, using the SurveyMonkey platform or a printed questionnaire applied before classes. Of the 400 students, 281 accepted to participate in the study and answered the questionnaire.

### Statistical analysis

We analyzed the data using the Statistical Package for the Social Sciences (SPSS) software version 21 and structural equation modeling (SEM) was performed using AMOS SPSS version 18. We excluded incomplete questionnaires (less than 80% of the items). As we intended to examine the new Portuguese version of the RMCCBS, we first performed the Exploratory Factor Analysis (EFA). In the EFA, we used Varimax rotation with Kaiser Normalization and Principal Axis Factoring extraction method [26]. Initially, the values ​​of the Kaiser-Meyer-Olkin (KMO) test (satisfactory values ​​above 0.500) and the Bartlett sphericity test were observed (significant values ​​acceptable, *p* < 0.05) [27]. We adopted the criteria of the latent root to estimate the number of factors to be retained. We analyzed the factorial loads obtained, observing the presence of low representativeness (factor load below 0.30), the factorial ambiguities (factorial loads similar to more than one factor) and empirical inconsistencies (factor empirically allocated to a different factor than theoretically predicted) [28].

We conducted confirmatory factor analysis (CFA), using structural equation modeling (SEM). We tested two alternative models: one following the best factorial solution achieved in the EFA and another following a single general factor [29]. We used the following indexes of adherence of the model: Comparative fit index (CFI), Goodness-of-fit index (GFI) and Tucker-Lewis Index (TLI). CFI, GFI and TLI ≤0.9. We considered the Bayesian Information Criterion (BIC) for Sample-size adjusted. The model with the lowest BIC was preferred. The root mean square error of approximation (RMSEA) was calculated. Values below 0.08 were considered acceptable [30]. Composite reliability analyzes were performed to evaluate the accuracy of the instrument. Values ​​above 0.70 were considered satisfactory [31].

### Ethical aspects

This study was approved by the Ethics Review Board of Bahiana School of Medicine and Public Health, CAEE number 57164216.1.0000.5544.

## Results

Of the 281 undergraduate medical students, 195 (69.3%) were female. Mean age of participants was 25.0 ± 2.6 years. The sample was distributed across the following semesters of the medical course: 9th (22.4%), 10th (29.2%), 11th (23.8%) and 12th (24.6%).

Table 1 shows the original English content of each item and the final version of the Portuguese translation. In the EFA, the KMO was 0.720 and the Bartlett sphericity test was significant (*p* < 0.001), demonstrating the adequacy of the sample and factorability of the correlation matrix.

**Table 1:** Items of the original role-modeling cost-conscious behaviors scale in English and its corresponding Portuguese version

ID	Original items	Translated items
1	Seek cost-effectiveness data to inform their clinical decision making	Buscar dados de custo-efetividade para compor as suas condutas clínicas
2	Initiate a conversation about costs of care when discussing treatment options	Iniciar uma conversa sobre custo dos cuidados em saúde ao discutir opções de tratamento
3	Refer a patient to a specialist because the patient wants it even when the physician does not believe a referral is indicated	Encaminhar um paciente a um especialista por vontade do paciente, mesmo quando o médico acredita que isso não seja necessário
4	Prescribe a brand name drug when an equivalent generic is available because a patient asks for the brand name drug specifically	Prescrever um medicamento de marca por pedido do paciente mesmo havendo genéricos equivalentes
5	Order a more expensive test or treatment because a patient requests it even if it offers only a small potential benefit compared to less costly alternatives	Solicitar um exame ou tratamento mais caro porque o paciente pediu, mesmo que o benefício potencial oferecido seja pequeno se comparado a alternativas menos caras.
6	Order numerous tests all at once rather than waiting to see the results of initial screening tests first	Solicitar vários exames de uma vez, ao invés de primeiro esperar para ver os resultados dos exames de rastreio (*screening*) primeiro
7	Repeat tests rather than attempt to obtain recently performed test results (e.g., by requesting a patient’s outside records)	Repetir exames em vez de tentar obter exames realizados recentemente (por exemplo: solicitando que o paciente traga resultados realizados em outros serviços)
8	Explain to a patient why a particular diagnostic test is not necessary	Explicar ao paciente porque um determinado teste diagnóstico não é necessário
9	Discuss costs of care with students or other members of the health care team when making patient care decisions	Discutir custos de cuidados em saúde com estudantes ou outros membros da equipe de saúde ao se tomar as decisões no cuidado com o paciente.
10	Ask a student or other member of the health care team to explain how a test result will affect patient management	Pedir a um estudante, residente ou outro membro da equipe de saúde que explique (ao paciente ou a equipe) como o resultado daquele exame diagnóstico irá interferir na conduta médica.
11	Criticize a student or resident for failing to order routine daily labs on a stable hospitalized patient	Advertir um estudante ou residente por não solicitar/sugerir exames laboratoriais diários de rotina em pacientes hospitalizados estáveis clinicamente.
12	Praise a student or resident for ordering a cost-effective diagnostic workup	Elogiar um estudante ou residente por solicitar/sugerir uma investigação diagnóstica mais custo-efetiva
13	Point out examples of waste in the health care system	Apontar exemplos de desperdício no sistema de saúde

As shown in Table 2, the latent root criterion supports solutions with up to four factors. However, the theoretical model for the scale contemplates only two dimensions. We decided to conduct the EFA into two factors. The rotated component matrix is shown in Table 3. The first factor grouped seven items corresponding to the dimension of role-modeling cost-conscious behaviors in health and the second factor grouped six items related to the dimension of health waste behaviors. High factor loads were obtained, varying between 0.416 and 0.701 in the first factor and between 0.346 and 0.670 in the second. No items were excluded.

**Table 2:** Factors retained by latent root criterion of the role-modeling cost-conscious behaviors scale in 281 undergraduate medical students, Salvador, Bahia, Brazil

**Factors**	**Eigenvalues**	**% Variance**	**% Cumulative**
1	3.104	23.878	23.878
2	2.062	15.859	39.737
3	1.215	9.349	49.086
4	1.071	8.241	57.327
5	.919	7.070	64.397
6	.866	6.658	71.055
7	.789	6.066	77.121
8	.704	5.416	82.537
9	.560	4.310	86.847
10	.498	3.830	90.677
11	.424	3.265	93.942
12	.411	3.164	97.106
13	.376	2.894	100.000

**Table 3:** Principal component analysis of the role-modeling cost-conscious behaviors scale in 281 undergraduate medical students, Salvador, Bahia, Brazil

Rotated Factor Matrix^a^	Factor*	
	1	2
Discuss costs of care with students or other members of the health care team when making patient care decisions	.701	
Initiate a conversation about costs of care when discussing treatment options	.657	
Seek cost-effectiveness data to inform their clinical decision making	.535	
Point out examples of waste in the health care system	.520	
Ask a student or other member of the health care team to explain how a test result will affect patient management	.477	
Praise a student or resident for ordering a cost-effective diagnostic workup	.456	
Explain to a patient why a particular diagnostic test is not necessary	.416	
Prescribe a brand name drug when an equivalent generic is available because a patient asks for the brand name drug specifically		.670
Order a more expensive test or treatment because a patient requests it even if it offers only a small potential benefit compared to less costly alternatives		.661
Order numerous tests all at once rather than waiting to see the results of initial screening tests first		.461
Repeat tests rather than attempt to obtain recently performed test results (e.g., by requesting a patient’s outside records)		.382
Refer a patient to a specialist because the patient wants it even when the physician does not believe a referral is indicated		.374
Criticize a student or resident for failing to order routine daily labs on a stable hospitalized patient		.346

^a^ Varimax rotation with Kaiser Normalization

*Factor loadings greater than 0.3

**Fig. 1:**
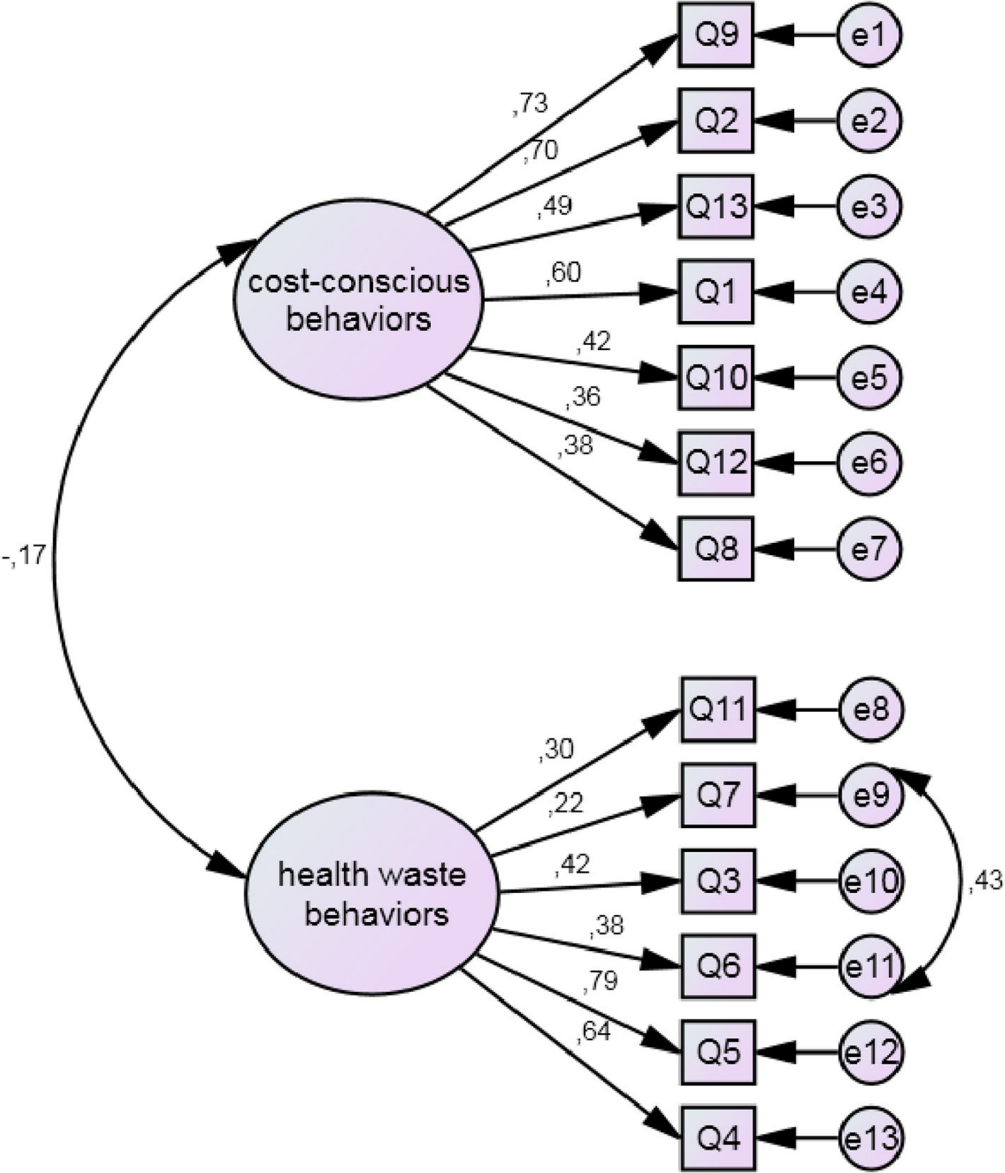
Confirmatory factor analysis for the role-modeling cost-conscious behaviors scale (chi square = 149,269; df = 63; *p* = 0.000; RMSEA = 0.070).

Table 4 presents the CFA results. The two-factor model showed higher adherence rates than the single factor alternative model. We observed the modification indices and included one parameter. Figure 1 presents the final model with factor loadings. The 13 item-scale was submitted to composite reliability analyzes, obtaining values of 0.813 and 0.761 for the role-modeling cost-conscious behaviors and the health waste behavior factors, respectively.

**Table 4:** Confirmatory factor analysis, using the structural equation modeling, of the role-modeling cost-conscious behaviors scale in 281 undergraduate medical students, Salvador, Bahia, Brazil

	X^2^	df	GFI	CFI	TLI	BIC	RMSEA (CI 90%)
Model 2 factors	201.614	64	.899	.783	.737	353.849	.088 (.074–.101)
Model 1 factor	345.347	65	.825	.559	.470	491.944	.124 (.111–.137)
Model 2 factors (re-specified)	149.269	63	.924	.864	.832	307.143	.070 (.056–.084)

*X*^2^ Chi-square, *Df* degrees of freedom, *GFI* goodness-of-fit index, *CFI* comparative fit index, *TLI* Tucker-Lewis Index, *BIC* Bayesian Information Criterion, *RMSEA* root mean square error of approximation, *CI* confidence interval

## Discussion

During the undergraduate medicine course, it is important to implement teaching and learning tools to encourage better cost-conscious care. Medical education literature present research in the area of physician’s attitudes and professionalism [11, 19–23], although only one study has developed a role-modeling cost-consciousness behaviors scale that assesses medical students’ perceptions of their teacher’s attitudes in the investigated area [8]. Our study has translated, culturally adapted and validated the RMCCBS scale in Brazil. To the best of our knowledge, there is no validation or publication of instruments that enable the evaluation of medical students’ perception of their teachers’ cost-conscious behaviors. Our study found that the RMCCBS scale is an important medical education tool and may help medical curricula implementation and better evaluation and intervention in medical learning environments.

For translation, cross-cultural adaptation and validation of the original English scale version, we adopted rigorous methodological approaches. We planned all the translation process from choosing the right translator and specialist profiles in order to enhance the quality of the translation, the back-translation and semantic and cultural validation. We also conducted a pilot test of the translated instrument, and it was evaluated by specialists before applying it among the undergraduate medical students.

We tested the psychometrical behavior of the scale-translated version, using exploratory and confirmatory factor analyses. The conduction of EFA and CFA in the same dataset should be avoided [29]. Although, we combined the use of these two techniques based in our purpose of exploring the latent variable structure of our dataset and then apply the previous theory of the original scale, conducting the CFA to test whether our dataset was suitable for the model [29]. As explained previously, factor analysis techniques aim to reduce a large number of items to a smaller number by observing the co-variations between items [27, 29]. As our study was based on a hypothetical model of distribution of the items, based on the original study, it would have been possible to conduct the confirmatory factor analysis alone. However, due to the lack of previous data on the validation of the model, we adopted a more conservative procedure, initially conducted for the EFA and then the CFA [29].

The exploratory factor analysis showed that the translated version of the scale is adequate to measure the perception of medical students’ exposure to physician role-modeling behaviors related to cost-conscious care. In agreement with the proposal of the original scale, the solution of two factors was adequate to represent the two phenomena in question. All tested items in this solution obtained satisfactory factor loads predicted model. There was no indication for item exclusions due to low representativeness or factorial ambiguity. The factor loading suggests the goodness-of-fit of the items to the scale content in a clear and precise way. The good quality of the items can be justified by the careful process of transcultural translation and adaptation of the scale, including semantic validation steps with medical students and specialists, allowing language adjustments and reducing biases.

In the CFA, the solution obtained in the EFA obtained better adhesion than the alternative model tested (single general factor). The adjustment indicators of the two-factor model were close to satisfactory. However, respecifications in the model proved to be necessary for its adherence to the desired parameters [30].

Among the respecifications indicated by the modification indices, the one that presented the greatest power of model improvement was the inclusion of a correlation parameter between the items “Order numerous tests all at once rather than waiting to see the results of initial screening tests first” and “Repeat tests rather than attempt to obtain recently performed test results (e.g. by requesting a patient’s outside records)”. Considering the proximity of item contents, we assumed the existence of a possible conceptual overlap, which would allow the exclusion of one of them, without compromising the representativeness of the factor. Taking into account that this is the first analysis of the psychometric properties of the instrument, we opted for the maintenance of both items and insertion of the parameter suggested improving the fit of the model, although this may be controversial in the literature [32]. After this insertion, the GFI and RMSEA the achieved desirable values ​​and the CFI and TLI scores were close to satisfactory [30]. Despite the possibility of including new parameters to make all indicators satisfactory, we opted to prioritize the parsimony of the model.

We conducted the composite reliability analyses to evaluate the internal consistency of the factors. Although the Cronbach’s alpha index is widely used as an indicator of consistency, researchers have pointed out some constraints. Some of these are because the extension of the test strongly affects the Cronbach’s alpha index and it is dependent on the number of items in the scale [33]. For this reason, we chose the composite reliability index that has been shown to be a more robust precision indicator in comparison to Cronbach’s alpha [31].

The composite reliability indexes were higher than 0.70 in both factors, evidencing the acceptable scale reliability. Although the indexes were satisfactory, the results are not excellent, especially for the second factor. The adoption of four-point Likert-type in the original scale may explain the reduction in scale reliability due to the small number of response categories, resulting in low variability and reliability [29]. However, we considered it important, for comparative purposes and validation, to adopt the same number of response categories as the original work.

In summary, the results suggest that the RMCCBS scale has satisfactory psychometric quality and is an adequate tool for measuring the perception of medical students’ exposure to two styles of behavior of their teachers: one that reflects the adoption and encouragement of cost-conscious behaviors in health and another one that indicates the adoption and incentive of waste behaviors in health. We would like to highlight that the two behavioral styles, represented by the factors, are not the extremes of the same continuum. It is possible, for example, that the same student presents high perceptions of both factors. As a result, we do not recommend the use of a single general score, but two scores: the first obtained by the average of items of factor 1 (cost-conscious behaviors in health) and the second according to the average of the items of factor 2 (health waste behaviors).

Our study has some limitations because it was conducted at a single Brazilian medical school. Local and cultural aspects may have influenced our results. We suggest the use of the instrument at medical schools and residency programs for curricula development, interventions in learning environments and evaluation of its results. We suggest and recommend the use of the RMCCBS at other Brazilian medical schools, and in different regions of the country which should provide opportunities to re-test the scale, increasing its validity and reliability.

## Conclusions

We conclude that the cost-conscious behaviors scale is a valid and reliable instrument for the evaluation of medical students’ perception of their teachers’ cost-conscious behaviors. The scale has good psychometric properties and represents a new research area in Brazil.

## Abbreviations

BIC: Bayesian Information Criterion
CFA: Confirmatory factor analysis
CFI: Comparative fit index
EFA: Exploratory factor analysis
GFI: Goodness-of-fit index
RMCCBS: Role-modeling cost-conscious behaviors scale
RMSEA: Root mean square error of approximation
SEN: Structural equation modeling
TLI: Tucker-Lewis Index
